# High Temperatures During the Seed-Filling Period Decrease Seed Nitrogen Amount in Pea (*Pisum sativum* L.): Evidence for a Sink Limitation

**DOI:** 10.3389/fpls.2019.01608

**Published:** 2019-12-20

**Authors:** Annabelle Larmure, Nathalie G. Munier-Jolain

**Affiliations:** Agroécologie, AgroSup Dijon, INRA, Univ. Bourgogne Franche-Comté, Dijon, France

**Keywords:** high temperatures, *Pisum sativum* L, Seed N amount, N partitioning, ^15^N labeling, seed-filling, plant N uptake

## Abstract

Higher temperatures induced by the on-going climate change are a major cause of yield reduction in legumes. Pea (*Pisum sativum* L.) is an important annual legume crop grown in temperate regions for its high seed nitrogen (N) concentration. In addition to yield, seed N amount at harvest is a crucial characteristic because pea seeds are a source of protein in animal and human nutrition. However, there is little knowledge on the impacts of high temperatures on plant N partitioning determining seed N amount. Therefore, this study investigates the response of seed dry matter and N fluxes at the whole-plant level (plant N uptake, partitioning in vegetative organs, remobilization, and accumulation in seeds) to a range of air temperature (from 18.4 to 33.2°C) during the seed-filling-period. As pea is a legume crop, plants relying on two different N nutrition pathways were grown in glasshouse: N_2_-fixing plants or NO_3_^−^-assimilating plants. Labeled nitrate (^15^NO_3_^−^) and intra-plant N budgets were used to quantify N fluxes. High temperatures decreased seed-filling duration (by 0.8 day per °C), seed dry-matter and N accumulation rates (respectively by 0.8 and 0.032 mg seed^−1^ day^−1^ per °C), and N remobilization from vegetative organs to seeds (by 0.053 mg seed^−1^ day^−1^ per °C). Plant N_2_-fixation decreased with temperatures, while plant NO_3_^−^ assimilation increased. However, the additional plant N uptake in NO_3_^−^-assimilating plants was never allocated to seeds and a significant quantity of N was still available at maturity in vegetative organs, whatever the plant N nutrition pathway. Thus, we concluded that seed N accumulation under high temperatures is sink limited related to a shorter seed-filling duration and a reduced seed dry-matter accumulation rate. Consequently, sustaining seed sink demand and preserving photosynthetic capacity of stressed plants during the seed-filling period should be promising strategies to promote N allocation to seeds from vegetative parts and thus to maintain crop N production under exacerbated abiotic constraints in field due to the on-going climate change.

## Introduction

Temperature is one of the main environmental factors explaining the variations in seed yield and quality in annual crop plants, especially legumes (Wheeler et al., 2000; Peng et al., 2004; Schlenker and Roberts, 2009; Asseng et al., 2011; Sita et al., 2017). The observed global increase in temperature (1.0°C of global warming above pre-industrial levels) is projected to continue by 0.2°C per decade due to past and ongoing emissions (including greenhouse gases) (IPCC, 2018). High temperatures are thus expected to be more frequent during the reproductive period of crops in temperate climate. They are already a major cause of the recent yields stagnation and projected decline due to the climatic change in Europe (Brisson et al., 2010; Supit et al., 2012; Trnka et al., 2012).

Pea (*Pisum sativum* L.) is an important annual legume crop grown in temperate regions for its high seed nitrogen (N) concentration. Including legumes in rotations leads to environmental benefits thanks to their unique capacity to acquire N via atmospheric N_2_ symbiotic fixation (Jensen and Hauggaard-Nielsen, 2003; Nemecek et al., 2008; Siddique et al., 2012). However, to extend the pea crop area in Europe, pea yield and seed protein concentration should be increased as well as their stability, especially in fluctuating climatic conditions (Siddique et al., 2012; Vadez et al., 2012).

Nitrogen yield (product of the yield and the seed N concentration) is a crucial characteristic at harvest in pea because seeds are a source of protein in animal and human nutrition. During the reproductive phase, N partitioning is the key process involved in the modulation of N yield. In most grain crops and, above all, in legumes, newly acquired N is generally low and insufficient to fulfill the high N demand of seeds, consequently endogenous N previously accumulated in vegetative parts is exported to seeds (Sinclair and Wit, 1976; Salon et al., 2001; Malagoli et al., 2005; Schiltz et al., 2005; Kichey et al., 2007; Barraclough et al., 2014). This remobilized N derives from the proteolysis of essential leaf proteins involved in photosynthesis, mostly Rubisco (Gregersen et al., 2008; Masclaux-Daubresse et al., 2008). The resulting decrease in leaf photosynthetic capacity may thus limit yield by shortening the duration of the seed-filling period (Sinclair and Horie, 1989; Munier-Jolain et al., 2008; Bueckert et al., 2015). Nitrogen remobilization not only affects yield, but also N yield since N remobilized from vegetative parts is the major contributor to seed N in most grain crops (Malagoli et al., 2005; Schiltz et al., 2005; Kichey et al., 2007; Araujo et al., 2012).

High temperatures affect plant phenology and carbon metabolism through various processes such as hastening reproductive development (Badeck et al., 2004; Barnabas et al., 2008; Bueckert et al., 2015; Sita et al., 2017), reducing photosynthesis (Guilioni et al., 2003; Kirschbaum, 2004; Sage and Kubien, 2007; Pimentel et al., 2013; Tacarindua et al., 2013), and reducing seed set (Guilioni et al., 2003; Djanaguiraman et al., 2013; Edreira and Otegui, 2013; Tacarindua et al., 2013; Bueckert et al., 2015). Conversely, impacts of high temperatures on assimilate partitioning remain unclear, especially concerning their effect on N remobilization to filling seeds. Some authors reported a decrease in N remobilization from vegetative parts to filling grain in response to heat stress in wheat (*Triticum aestivum* L.) (Tahir and Nakata, 2005) and in rice (*Oryza sativa*) (Ito et al., 2009). On the contrary, other authors suggest that high temperatures increase N remobilization from vegetative organs to seeds causing an acceleration of senescence (Spiertz, 1977; Morison and Lawlor, 1999; Masclaux-Daubresse et al., 2008; Zhao et al., 2011; Wu et al., 2012). Moreover, high temperatures may also affect N uptake of legumes (mainly acquired via N_2_ fixation), but unfortunately little is known about temperate legume crops (Bordeleau and Prevost, 1994).

Further investigations are thus needed to improve the understanding of the effect of high temperatures on N assimilate partitioning during the seed-filling period and to quantify the impact on seed N yield in legumes. For this purpose, the present study therefore assessed the response of seed dry matter and N fluxes at the whole-plant level (seed N accumulation, N remobilization, plant N uptake, and N amount variation in vegetative organs) to contrasting temperature ranging from permissive to heat stress during the seed-filling period of pea (Guilioni et al., 2003). We compared N_2_-fixing and NO_3_^−^-assimilating plants, the first being more representative of field conditions while the later allow the use of a ^15^NO_3_^−^-labeled nutrient solution to assess N fluxes.

## Materials and Methods

### Plant Material and Growth Conditions

Three different glasshouse experiments (Exp. 1, 2, and 3) were conducted. One single line of spring dry pea (cv. Baccara) has been used, all plants were genetically identical. Baccara characteristics are described in Bourion et al. (2002a; 2002b). Pea seeds were sown in 5 L pots at a density of eight plants per pot. Pots were filled with a 1:1 (v/v) mixture of sterilized attapulgite and clay balls (diameter 2–6 mm) in Exp. 1 and 3 or with a mixture of 1/6 vermiculite, 1/3 siliceous sand, and 1/2 clay balls (diameter 2–6 mm) in Exp. 2. After seedling establishment the plants were thinned to the four most homogeneous per pot. Plant N nutrition relied exclusively on NO_3_^−^ assimilation in Exp. 1 and 2 due to the high nitrate availability of the nutrient solution (14 meq NO_3_^−^, P, K, and micronutrients; **Table S1**). In Exp. 3, pea plant N nutrition relied exclusively on N_2_ fixation due to a nutrient solution without nitrate (0 meq NO_3_^−^, P, K, and micronutrients; **Table S1**) and an inoculation. Seedlings were inoculated with 1ml of Rhizobacterium leguminosarum bv. Vicieae, strain P221 (MIAE01212, 10^8^ bacteria per plant), the strain usually used in the laboratory because of its good efficiency in particular with cv. Baccara (Voisin et al., 2013).

Photosynthetic active radiation was provided to the plants by day light and mercury lamps (MACS 400 W; Mazda, Dijon, France) with a 14-h day length. The air temperature was recorded every 5 min in order to calculate the mean daily temperature. Prior to the different temperature treatments, the glasshouse temperature was maintained at a day/night temperature of 24/16°C.

### Temperature Treatments During the Seed-Filling Period

The experiments aimed at testing the effect of temperature during the seed-filling period. As peas are indeterminate plants with a sequential flowering up the stem leading to a wide heterogeneity of seed developmental stages, the temperature treatments started at the beginning of seed filling of the last reproductive node (BSL) and ended when seed physiological maturity was reached at the whole-plant level, as described by Larmure et al. (2005).

At BSL, different sets of pots were randomly transferred to glasshouses maintained at the different day/night temperatures until plant physiological maturity. The air temperature treatments tested in Exp. 1, 2, and 3 ranged approximately from 20/16°C to 35/31°C day/night (**Table 1**). In Exp. 1 and 2 monitoring NO_3_^−^-assimilating plants, respectively four and three day/night temperatures were chosen in order to form a range of seven temperatures. In Exp. 3 monitoring N_2_-fixing plants, four day/night temperatures were chosen in order to form a temperature range similar to that tested for ${\text{NO}}_{3^{-}}$-assimilating plants.

**Table1:** Glasshouse experiments characteristics and seed number, individual seed dry weight, seed N concentration and amount, and vegetative organs N concentration at maturity.

	N nutrition pathway	Mean temperature during the seed-filling period			Seed number at maturity	Individual seed dry weight at maturity	Seed N concentration at maturity	Seed N amount at maturity	Vegetative organs N concentration at maturity
		Day	Night	Mean	(plant^−1^)	(mg)	(mg g^−1^)	(mg plant^−1^)	(mg g^−1^)
		(°C)	(°C)	(°C)					
Exp. 1	NO_3_**^−^**	20.3 (±0.2)	15.9 (±0.2)	18.4 (±0.2)	18.1a	289a	40.2a	210a	23.5
	Assimilation	25.0 (±0.1)	20.7 (±0.1)	23.2 (±0.1)	16.6a	261a	42.7b	185b	26.7
		29.4 (±0.5)	26.3 (±1.2)	28.1 (±0.6)	18.7a	199b	46.8c	174bc	41.2
		34.5 (±0.5)	31.4 (±0.5)	33.2 (±0.4)	15.1a	169c	47.4c	121de	43.8
Exp. 2	NO_3_**^−^**	24.1 (±0.5)	18.3 (±0.4)	21.8 (±0.6)	9.1b	305a	38.4a	107ef	23.9
	Assimilation	28.0 (±1.7)	22.8 (±1.5)	25.8 (±1.5)	8.9b	275a	40.7ab	100f	27.8
		28.9 (±1.9)	23.9 (±2.3)	26.8 (±1.9)	8.6b	258a	43.2abc	96f	29.3
Exp. 3	N_2_	21.8 (±0.6)	17.2 (±0.5)	19.9 (±0.5)	18.4a	232ab	40.6ab	173bc	17.6
	Fixation	27.8 (±1.1)	23.7 (±0.1)	26.1 (±0.6)	17.4a	210b	41.3ab	150cd	16.1
		30.3 (±0.9)	26.3 (±0.5)	28.6 (±0.8)	18.1a	173bc	43.4ab	135de	16.3
		32.8 (±1.7)	29.5 (±0.4)	31.3 (±1.5)	16.5a	147c	45.6bc	110ef	19.9

Pea plants were exposed to temperature treatments during the seed-filling period, i.e. from the beginning of seed filling of the last reproductive node (BSL) to plant maturity. Mean temperatures during the seed-filling period (with standard error) were assessed as the average of the daily air temperatures observed from BSL to maturity (14-h day length). Values with the same letter are statistically not different at P = 0.05.

The temperatures were modified gradually during two acclimatization days to reach the temperature objectives of each treatment. All temperature treatments are described in **Table 1** including the average of mean air temperatures actually observed in the glasshouses (ranging from 18.4 to 33.2°C). Plants were maintained at the maximum soil water capacity by providing non-limiting water availability with an automatic watering system.

### Plant Sampling and Measurements

Prior to the different temperature treatments, seed water concentration was destructively measured at each node twice a week to assess the date of BSL.

For each temperature treatment, randomly chosen pots were harvested: (1) at the beginning of the temperature treatment, (2) during the temperature treatment, and (3) after plant physiological maturity (three pots per treatment for Exp. 1 or five pots per treatment for Exp. 2 and 3). At each sampling date, seeds, leaves, stems, pod walls, and roots were collected separately. Dry matters, seed number, and water concentration were determined as described by Larmure et al. (2005).

In Exp. 1 and 2, total N concentrations and ^15^N enrichments were determined using a dual inlet mass spectrometer coupled with a CHN analyzer (Sercon, ANCA-GSL-2020). In Exp. 3, total N concentrations were determined with an elemental analyser (Carlo Erba).

### Determination of N Fluxes

Nitrogen fluxes (seed N accumulation, endogenous-N remobilization, plant exogenous-N uptake, and N amount variation in vegetative organs) were expressed in mg seed^−1^ day^−1^. This unit is adequate to depict N partitioning to seeds in plants, because the individual seed N accumulation rate depends on N available per seed (N from endogenous-remobilization and exogenous sources) (Lhuillier-Soundélé et al., 1999; Larmure and Munier-Jolain, 2004). Moreover, this unit allows to compare N fluxes in plants differing in seed number and vegetative parts biomass.

#### Plant ^15^N Labeling and Calculation of N Fluxes for ${\text{NO}}_{3^{-}}$-Assimilating Plants

^15^N labeling sessions with NO_3_^−^-assimilating plants (Exp. 1 and 2) were used to distinguish the remobilization of endogenous-^14^N stored before labeling from the exogenous-^15^N uptake supplied by ^15^NO_3_^−^ nutritive solution with 5% ^15^N APE (atom percent excess) enrichment. Successive 3-day labeling sessions were conducted during the temperature treatments as described by Schiltz et al. (2005). Homogenous groups of six pots for Exp. 1 or 10 pots in Exp. 2 were constituted and randomly used for the each labeling session. The first labeling session began at the end of the two acclimatization days. In Exp. 1, three successive labeling sessions were conducted for all temperature treatments, except for the warmest treatment permitting only two labeling sessions due to an earlier physiological maturity. In Exp. 2, two successive labeling sessions were conducted for all temperature treatments. At the beginning of each session, unlabeled control pots were harvested (three pots for Exp. 1 or five pots for Exp. 2). During the session, labeled pots were supplied during three days with the ^15^NO_3_^−^ nutritive solution and harvested (three pots for Exp. 1 or five pots for Exp. 2).

For ${\text{NO}}_{3^{-}}$-labeled assimilating plants, N fluxes were assessed using the data of the labeling sessions. Rates of plant N uptake, seed N accumulation, endogenous-N remobilization to filling seeds, and variation of N amount in each vegetative organ during a labeling session were calculated using the total N concentrations and the ^15^N enrichment of the labeling nutrient solution (5 %) as described by Schiltz et al. (2005). Each N flux value represents the mean value of the two or the three 3-day labeling sessions. Values resulted from the measurement of three (Exp. 1) or five (Exp. 2) biological replicates, each consisting of one pot with four plants.

#### Calculation of N Fluxes for N_2_-Fixing Plants

For unlabeled N_2_-fixing plants (Exp. 3), rates of plant N uptake, seed N accumulation, and variation of N amount in each vegetative organ were assessed as the linear regressions coefficients of each variable (plant N, seed N, and vegetative organ N amounts, respectively) *v*. time (expressed in days). Values resulted from the measurement of five biological replicates, each consisting of one pot with four plants. Endogenous-N remobilized to filling seeds could not be determined in Exp. 3 using unlabeled N_2_-fixing plants.

### Statistical Analysis

The experiments were conducted with completely randomized design with three (Exp. 1) or five (Exp. 2 and 3) biological replications. Each biological replication consisting of one pot with four plants (one single line, *cv*. Baccara). Data were analyzed using SigmaPlot® 12 (Systat Software, Inc.). All data obtained were subjected to analysis of variance. Differences at *P* ≤ 0.05 were considered significant.

## Results

### Seed Number, Individual Seed Dry Weight, Seed N Amount, and Seed N Concentration At Maturity

Seed number per plant at maturity was not significantly different among temperature treatments within an experiment (**Table 1**). Seed N amount at maturity and individual seed dry weight decreased in response to increasing temperatures in all three experiments (**Table 1**). On the contrary, seed N concentration increased with the increase in temperature in all experiments (**Table 1**). These changes of seed characteristics at maturity were significant for Exp. 1 and 3, that explored a wider range of mean daily air temperature during the seed-filling period than Exp. 2 (**Table 1**).

Seed number per plant at maturity was significantly different between experiments: it was lower in Exp. 2 than in Exp. 1 and 3 (**Table 1**), as was total seed dry matter (**Table S2**). And thus, seed N amount at maturity was also lower in Exp. 2 than in Exp. 1 and 3 (**Table 1**).

### Response of Seed Dry Matter Accumulation and Seed N Accumulation to the Increase in Temperature

**Figure 1:**
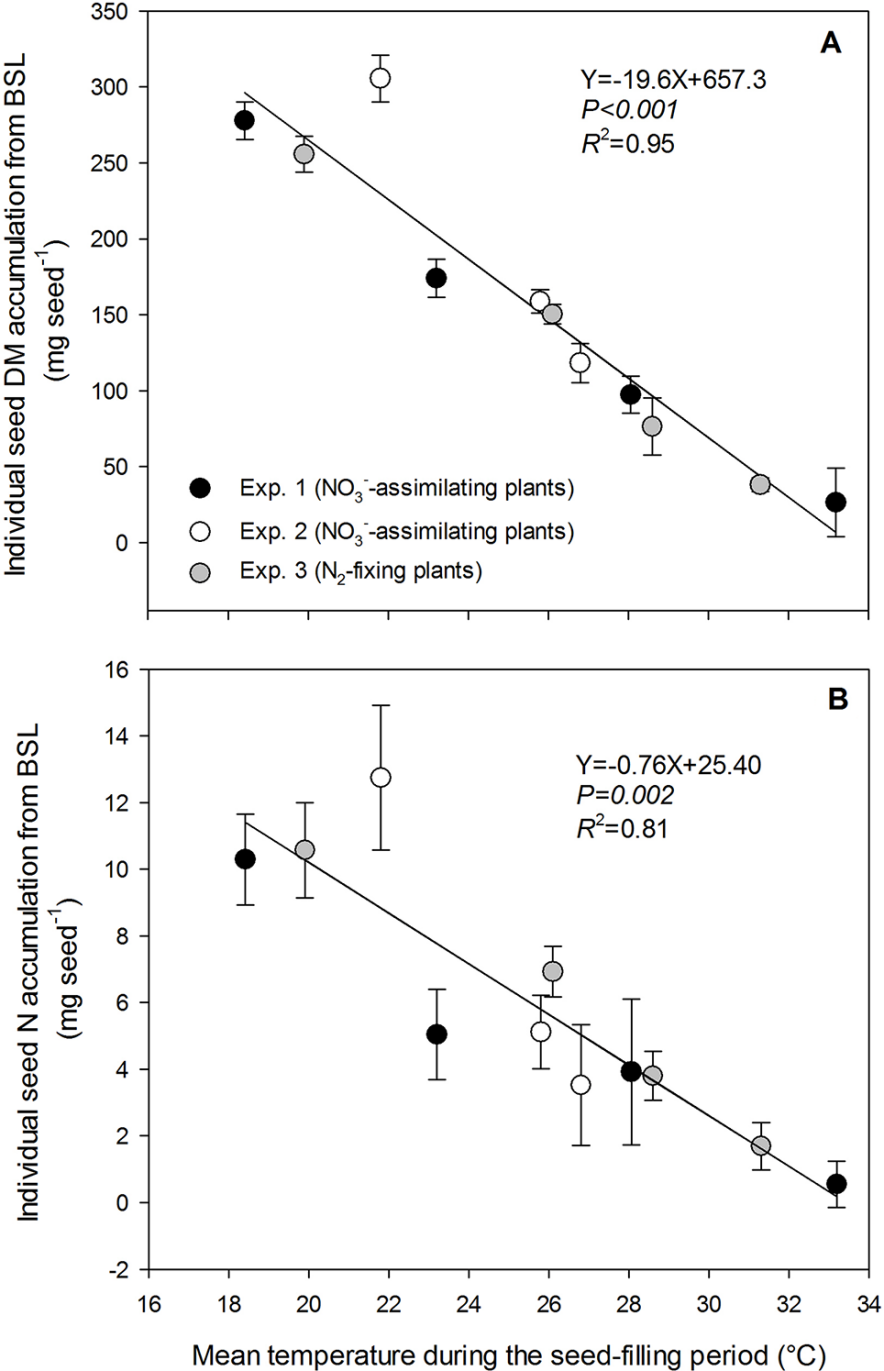
Decrease in individual seed dry matter accumulation **(A)** and individual seed nitrogen accumulation **(B)** with increasing temperature of the treatments during the seed-filling period. Pea plants were exposed to temperature treatments from the beginning of seed filling of the last reproductive node (BSL) to plant maturity. The vertical bars represent SE (when larger than symbol). The data were fitted with a linear regression.

**Figure 2:**
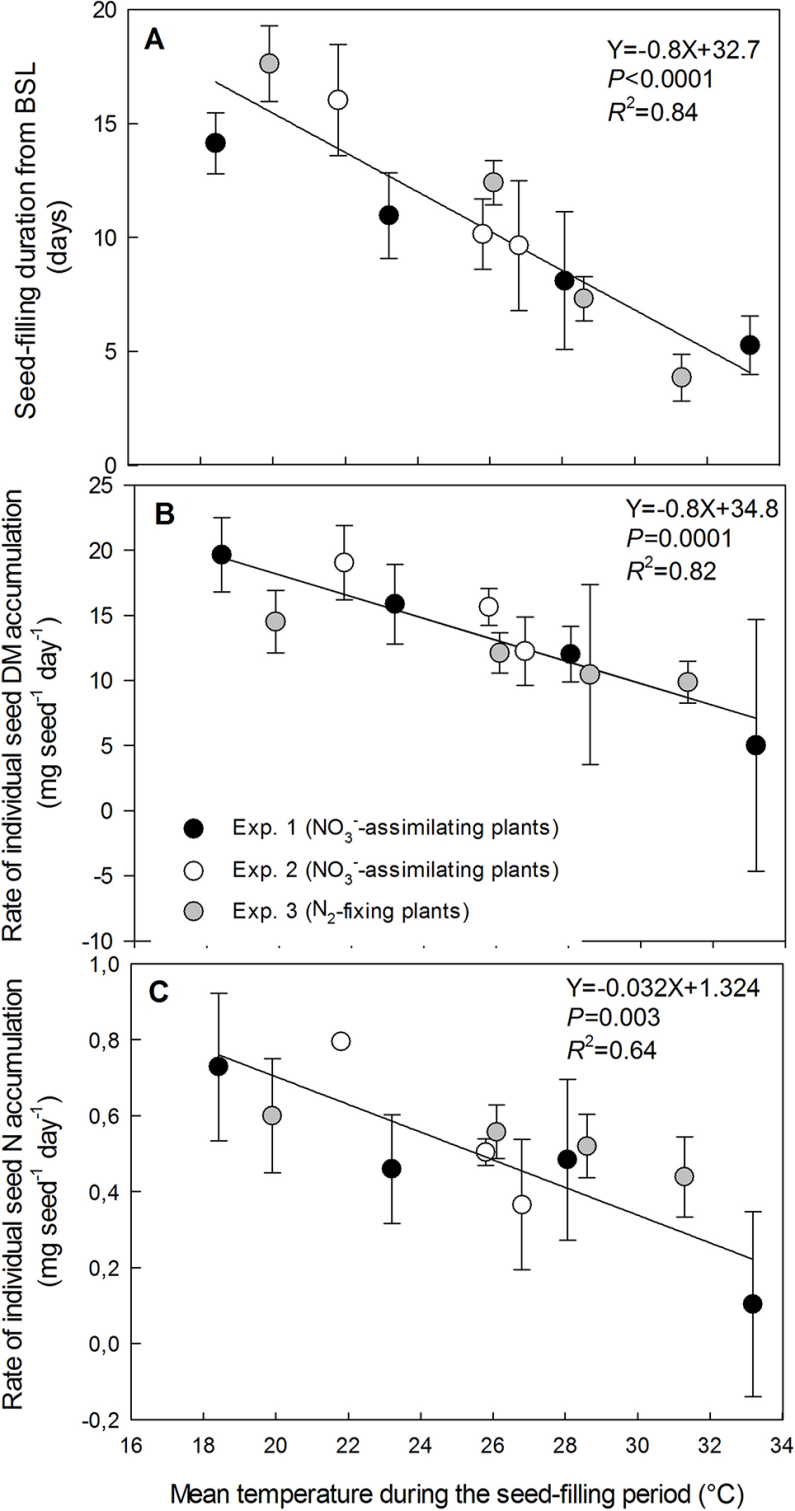
Decrease in seed-filling duration **(A)**, the rate of individual seed dry-matter accumulation **(B)** and the rate of individual seed nitrogen-accumulation **(C)** with increasing temperature of the treatments during the seed-filling period. The vertical bars represent SE (when larger than symbol). The data were fitted with a linear regression.

Individual seed dry matter accumulation during the seed-filling period decreased linearly with increasing temperature for both NO_3_^−^-assimilating and N_2_-fixing plants (data from the three experiments gathered) by 19.6 mg seed^−1^ per °C, from 227.8 mg seed^−1^at 18.4°C to 26.5 mg seed^−1^ at 33.2°C (*R*^2^ = 0.95) (**Figure 1A**). Individual seed dry matter accumulation was assessed as the product of the seed-filling duration and the rate of seed dry matter accumulation during the temperature treatments. Both variables significantly decreased with increasing temperature for the three experiments and for both plant N nutrition pathways (**Figures 2A**, **B**). The seed-filling duration was reduced progressively by 0.8 day for each additional °C (**Figure 2A**). Similarly, the rate of seed dry matter accumulation decreased progressively by 0.8 mg seed^−1^ day^−1^ per °C from 19.8 mg seed^−1^ day^−1^ at 18.4°C to 5 mg seed^−1^ day^−1^ at 33.2°C (**Figure 2B**).

Individual seed N accumulation during the temperature treatments decreased linearly with increasing temperature for both NO_3_^−^-assimilating and N_2_-fixing plants (data from the three experiments gathered) by 0.76 mg seed^−1^ per °C from 10.3 mg N seed^−1^at 18.4°C to 0.55 mg N seed^−1^ at 33.2°C (*R*^2^ = 0.81) (**Figure 1B**). Seed N accumulation was assessed as the product of the seed-filling duration and the rate of seed N accumulation during the temperature treatments. Both variables significantly decreased with increasing temperature from 18.4°C to 33.2°C, for the three experiments and both plant N nutrition pathways (**Figures 2A**, **C**). The rate of seed N accumulation decreased progressively by 0.032 mg seed^−1^ day^−1^ per °C from 0.73 mg seed^−1^ day^−1^ at 18.4°C to 0.10 mg seed^−1^ day^−1^ at 33.2°C (**Figure 2C**).

### Effect of High Temperatures on the Remobilization of Endogenous-N to Filling Seeds by NO_3_^−^-Assimilating Plants

**Figure 3:**
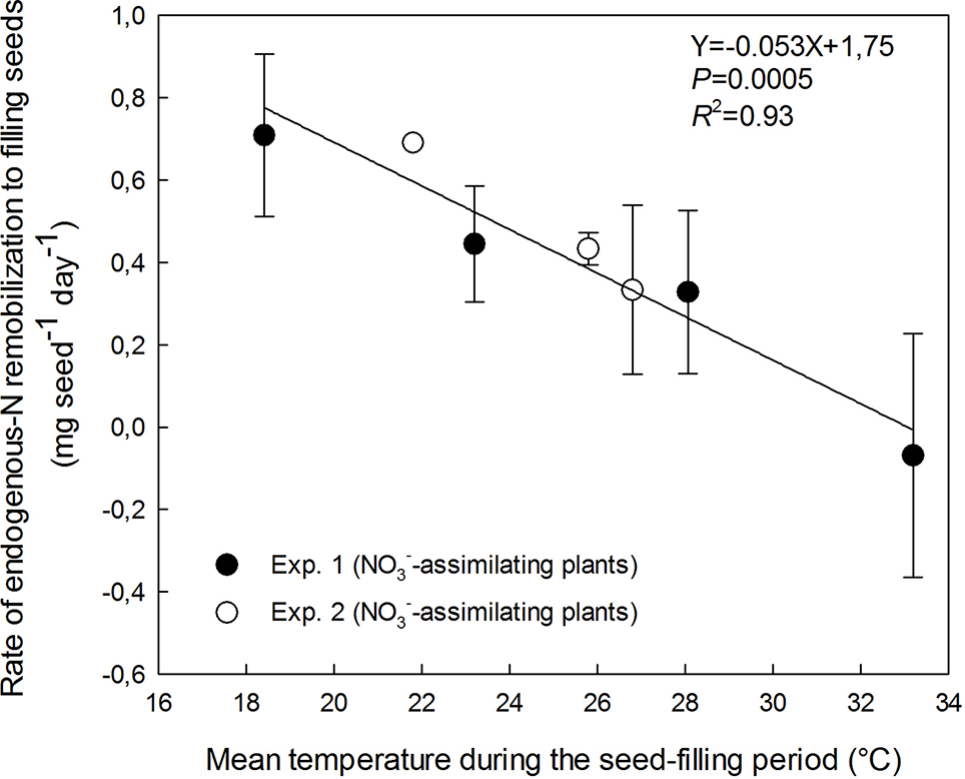
Decrease in the rate of endogenous-N remobilization from vegetative parts (roots, pod walls, stems, and leaves) to filling seeds with increasing temperature of the treatments during the seed-filling period. The vertical bars represent SE (when larger than symbol). The data were fitted with a linear regression.

Endogenous-N remobilization to filling seeds was measured on labeled NO_3_^−^-assimilating plants in Exp. 1 and 2. The contribution of remobilized N to the rate of seed N accumulation exceeded 82 % in both experiments with NO_3_^−^-assimilating plants (Exp. 1 and 2) for all temperatures (**Figures 2B** and **3**). The temperature increase dramatically decreased the rate of N remobilization to filling seeds from 0.71 mg seed^−1^ day^−1^ at 18.4°C to 0 at 33.2°C (**Figure 3**). The detrimental effect of increasing temperature suggests a full stop of N remobilization at a temperature around 33°C (intersection of regression and X-axis in **Figure 3**).

### Effects of High Temperatures on the Plant N Uptake by NO_3_^−^-Assimilating and N_2_-Fixing Plants

The rate of plant N uptake during the seed-filling varied between 0.11 and 0.64 mg seed^−1^ day^−1^ whatever the plant nutrition pathway. The variation range of the plant N uptake rate for N_2_-fixing plants was included in the variation range for NO_3_^−^-assimilating plants.

**Figure 4:**
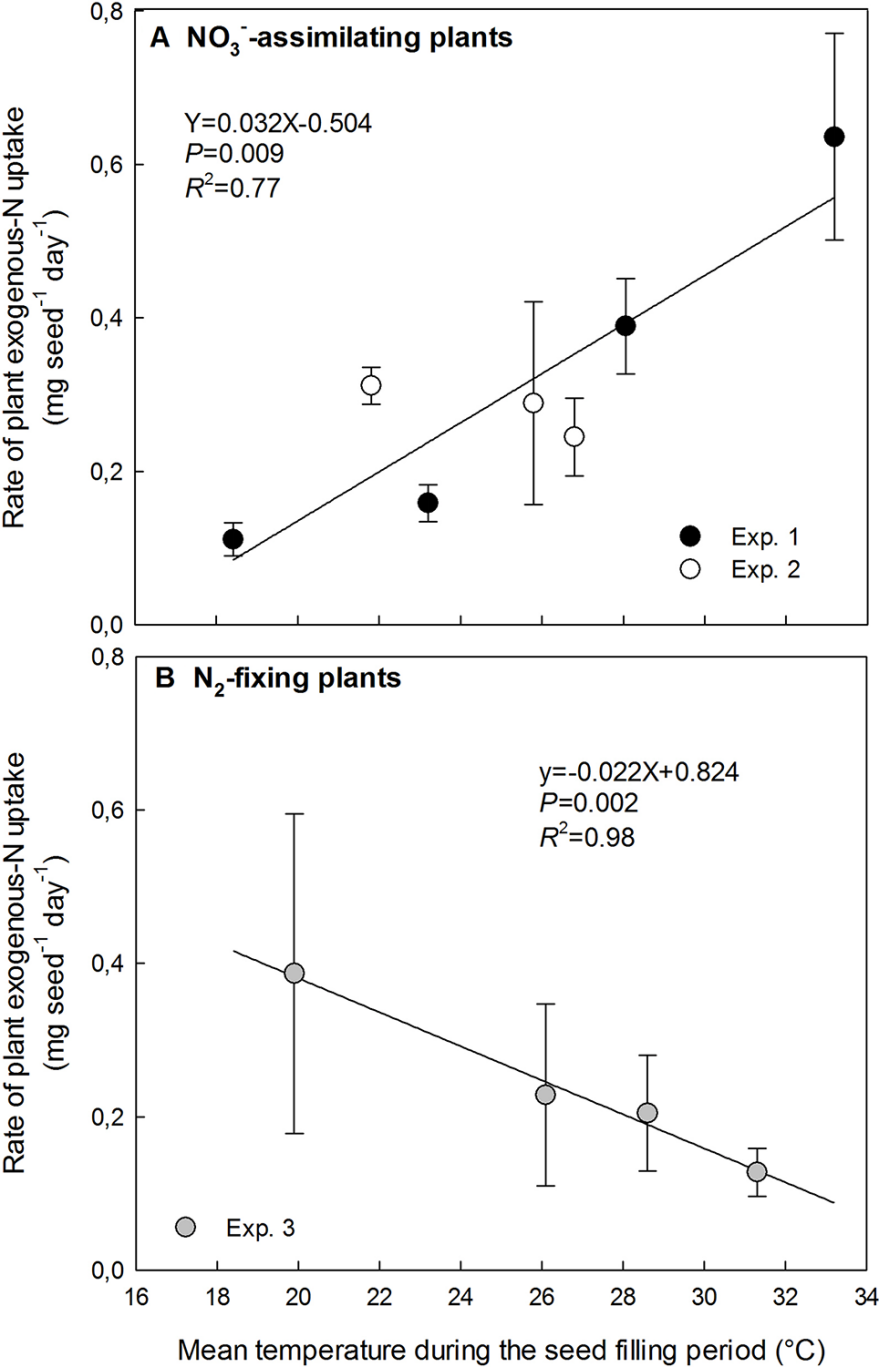
Opposite responses to temperature increase of exogenous-N uptake rate in plants during the seed-filling period for NO_3_**^−^**-assimilating plants **(A)** and N_2_-fixing plants **(B)**. The vertical bars represent SE. The data were fitted with a linear regression.

The rate of plant N uptake relying exclusively on NO_3_^−^ assimilation significantly increased from 0.11 mg seed^−1^ day^−1^ at 18.4°C to 0.64 mg seed^−1^ day^−1^ at 33.2°C (**Figure 4A**). Plant N uptake was not significantly modified by the small range of increasing temperature from 21.8 to 26.8°C in Exp. 2, while it increased linearly with increasing temperature from 18.4 to 33.2°C in Exp. 1 (**Figure 4A**).

Conversely, for N_2_-fixing plants in Exp. 3 the temperature increase significantly decreased the rate of N uptake in plants following a linear relationship from 0.39 mg seed^−1^ day^−1^ at 19.9°C to 0.13 mg seed^−1^ day^−1^ at 31.3°C (**Figure 4B**).

### Effects of High Temperatures on the Variation of the N Amount Within the Different Vegetative Organs During the Seed-Filling Period of NO_3_^−^-Assimilating and N_2_-Fixing Plants

**Figure 5:**
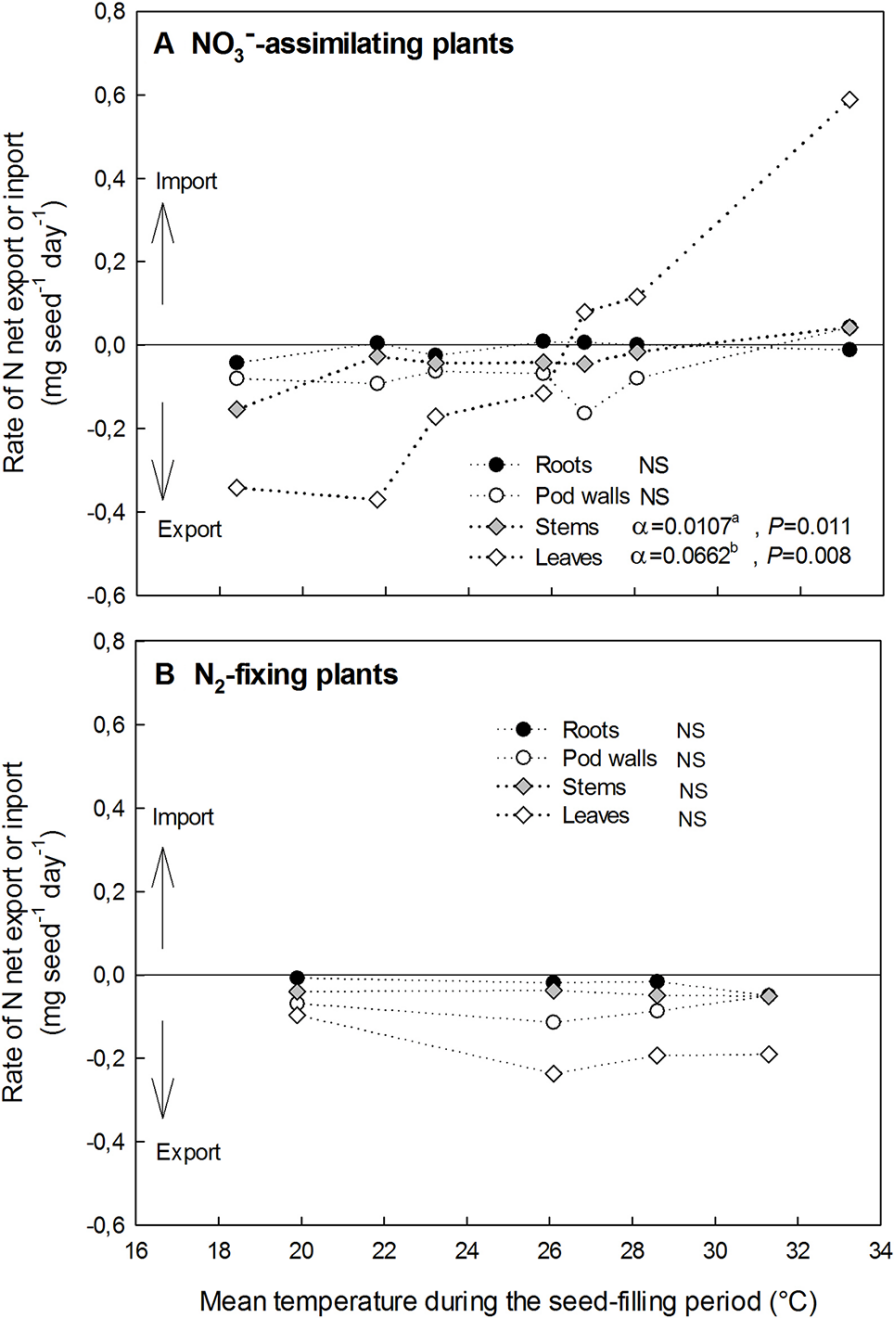
Impacts of the temperature increase on the rate of net N export or import in vegetative organs (roots, pod walls, stems, and leaves) during the seed-filling period for NO_3_^−^-assimilating plants **(A)** and N_2_-fixing plants **(B)**. α represents the linear regressions correlation coefficient of the net N export (or import) in each vegetative organ v. temperature. NS, not significantly different from 0 (*P* < 0.05). Values with different letters are statistically different at *P* = 0.05.

A net export of N represents a decrease in the N amount of a vegetative organ during the temperature treatment application through the seed-filling period, while a net import represents an increase in the N amount (**Figure 5**).

Considering NO_3_^−^-assimilating plants (Exp. 1 and 2), the effect of temperature on rates of the N amount variation during the seed-filling period was significant only in leaves and to a lesser extent in stems (**Figure 5A**). In the leaves, N fluxes switched from N export to N import approximately above 26.3°C (**Figure 5A**). At the lowest temperature (18.4°C) leaves and stems respectively exported 0.34 and 0.15 mg seed^−1^ day^−1^, while at the highest temperature (33.2°C) leaves and stems respectively imported 0.59 and 0.04 mg seed^−1^ day^−1^ (**Figure 5A**). Thus, the rate of the N amount variation during the seed-filling period in leaves was by far the most responsive to temperature among vegetative organs in NO_3_^−^-assimilating plants (**Figure 5A**).

Considering N_2_-fixing plants (Exp. 3), all vegetative organs presented a net export of N whatever the temperature (**Figure 5B**). The temperature increase (from 19.9 to 31.3 °C) had no significant effect on the rate of the N export whatever the vegetative organ of N_2_-fixing plants (**Figure 5B**).

At maturity, N concentrations of vegetative organs (roots, pod walls, stems, and leaves) were above 16 mg g^−1^, for both NO_3_^−^-assimilating and N_2_-fixing plants and whatever the temperature treatment (**Table 1**).

## Discussion

The present study quantifies and explains, for the first time, the effects of high temperatures on N partitioning to filling seeds in pea, an annual legume crop. Plants differing in seed number between experiments allow us to assess trends representative of various field conditions. The wide range of mean air temperature explored (from 18.4 to 33.2°C) is representative of the present and future climatic conditions expected in field during the seed-filling period of most annual crops in Western Europe (June-July): mean monthly temperatures above 18°C and an increase in the frequency, intensity, and duration of heat waves (Christensen et al., 2007; Vliet et al., 2012; Xu et al., 2012). This temperature range was similar for the two plant N nutrition pathways tested: 19.9 to 31.3°C for NO_3_^−^-assimilating plants allowing to measure endogenous fluxes and 18.4 to 33.2°C for N_2_-fixing plants, more representative of field conditions. Temperature treatments started when all seeds had begun to fill. At this stage, pea plants had no longer the possibility to adjust the number of seed sinks to assimilate availability as earlier in their development (Ney et al., 1993). Indeed, seed number per plant at maturity was equal for all temperature treatments within an experiment.

### Decrease in Seed Dry Matter and N Accumulation With Increasing High Temperature, Resulting Effects on Seed N Concentration and N Yield

The rate of individual seed dry matter accumulation and seed-filling duration in pea were reduced by 0.8 mg seed^−1^ day^−1^ and 0.8 days, respectively, for each additional °C of mean temperature from 18.4 to 33.2°C. Therefore, individual seed weight decreased with increasing temperature. These results are consistent with previous reports of a reduction in seed weight at high temperatures due to a decrease in the rate of seed fill and an abbreviated seed-filling duration (Singletary et al., 1994; Kim et al., 2011; Bueckert et al., 2015).

Our study demonstrates that seed N accumulation was also reduced by 0.76 mg seed^−1^ day^−1^ for each additional °C of mean temperature from 18.4 to 33.2°C, for both NO_3_^−^-assimilating and N_2_-fixing plants. Results showed that, whatever the plant N nutrition pathway, the decrease of seed N accumulation with increasing temperature was due to the reduction of both the rate of individual seed N accumulation and the seed-filling duration. The rate of individual seed N accumulation progressively decreased by 0.032 mg seed^−1^ day^−1^ for each additional °C temperature from 18.4 to 33.2°C. Therefore the amount of N accumulated in seeds significantly decreased with increasing temperatures.

Seed N concentration at maturity is the ratio of seed N and seed dry matter accumulation rates during the seed-filling period. Our results demonstrate that the decrease of the individual seed N rate with increasing high temperatures was lower than that of the individual seed dry matter rate (0.032 and 0.8 mg seed^−1^, respectively). Thus seed N concentration increased with increasing high temperatures. This result is consistent with previous reports of higher seed N concentration when temperatures rise during the seed-filling period (Karjalainen and Kortet, 1987; Tashiro and Wardlaw, 1991; Wardlaw and Wrigley, 1994; Larmure et al., 2005; Farooq et al., 2018).

In Europe, the current and projected warming rate in summer (June to August) is between 4.5 and 6.8°C/century, higher than for other seasons (Rowell, 2005; Xu et al., 2012; Terray and Boe, 2013). Consequently, the on-going climate warming has caused and will continue to cause severe seed N yield losses in pea without adaptation strategies. From our study, it can be expected that at the field scale, seed N yield in pea could decrease by 1.8 gN m^−2^ for each additional °C of mean temperature during the seed-filling period, considering 2,400 seed m^−2^. From the perspective of French pea production, it represents more than 13 % loss of recent seed N yield (~13.8 gN m^−2^ calculated with the mean yield and seed N concentration from 2013 to 2017: respectively 3.83 t·m^−2^ and 36.2 mgN·g^−1^ ; UNIP and ARVALIS, 2013, 2014; Terres Inovia and Terres Univia, 2015, 2016, 2017). Our study enables the identification of plant mechanisms involved in these seed N yield losses in order to provide levers for improving varieties tolerating heat stress.

### Nitrogen Sources Availability Does Not Explain the Decrease in Seed N Amount With Increasing High Temperature

Nitrogen for pea seeds comes from two sources: current plant N uptake and N remobilization from vegetative organs (Lhuillier-Soundélé et al., 1999; Schiltz et al., 2005). Nitrogen availability from plant sources is known to determine seed N accumulation (Lhuillier-Soundélé et al., 1999; Martre et al., 2003; Larmure and Munier-Jolain, 2004; Kinugasa et al., 2012) . However, our results contradict the possibility of a decrease in seed N accumulation at high temperatures resulting of a limitation in N supply.

Indeed, plant NO_3_^−^ assimilation provides higher N availability under high temperatures (with non-limiting water availability) as plant N uptake of NO_3_^−^-assimilating plants significantly increased with increasing temperature by 0.032 mg seed^−1^ day^−1^ for each additional °C temperature. NO_3_^−^ assimilation may have been enhanced by the increase in plant transpiration with increasing temperature under our no-limiting water conditions, because the transport of water and N solutes from roots to shoots is driven by the evaporative loss of water (Salon et al., 2011). Indeed, the transpiration of well-watered plants is expected to increase by 1–5% for each additional °C temperature between 5 and 35°C (Kirschbaum, 2004). Contrary to NO_3_^−^ assimilation, plant N_2_ fixation was reduced under high temperatures: plant N uptake of N_2_-fixing plants decreased with increasing temperature by 0.022 mg seed^−1^ day^−1^ for each additional °C temperature. High temperatures may decrease N_2_-fixation efficiency by affecting nitrogenase activity and/or nodule longevity (Bordeleau and Prevost, 1994; Hungria and Vargas, 2000), as no nodule production occurs during the seed-filling period of N_2_-fixing plants (Voisin et al., 2003; Bourion et al., 2007).

Despite the opposite effect of increasing temperature on plant N uptake acquired via N_2_ fixation or NO_3_^−^ assimilation, a lot of N was still available at maturity in vegetative organs (leaves, stems, pod walls, and roots), whatever the plant N nutrition pathway and the temperature treatment. Concentrations of vegetative organs at maturity were all above 16 mg g^−1^, much higher than the threshold of non-remobilizable N concentration (Larmure and Munier-Jolain, 2004). This result suggests that the shorter duration of seed-filling at high temperature was not due to a reduction of photosynthetic activity caused by N remobilization from vegetative organs to seeds. Indeed, the present study using ^15^NO_3_^−^-labeled N source clearly demonstrates a gradual limitation of the rate of endogenous-N remobilization from vegetative organs to filling seeds above 18.4°C. N remobilization was nevertheless the major contributor to the N filling of pea seeds whatever the temperature, consistently with the previous observations at non-stressing temperatures in oilseed rape (*Brassica napus*) and in pea (Malagoli et al., 2005; Schiltz et al., 2005).

### Sink Strength Determines Plant N Fluxes to Filling Seeds Under Heat Stress Conditions

Our results demonstrate a sink limitation of seed N accumulation by high temperatures (from 18.4 to 33.2°C). Actually, additional plant N uptake in NO_3_^−^-assimilating plants at high temperature provided by the xylem was never allocated to seeds but stored in leaves and to a lesser extent in stems. This findings are in line with the observation that the majority of seeds N intake is attributable to phloem (Pate and Hocking, 1978). This hypothesis of sink limitation at high temperature is consistent with (1) the shorter duration of seed-filling with increasing temperature observed in our study, that leads to a progressive premature reduction of seed sink; (2) the decrease of the individual seed dry matter accumulation rate with increasing temperature that reduces seed sink; and (3) previous studies reporting a decrease in photoassimilates translocation to filling seeds at high temperatures due to reduced sink activity rather than source activity (Ito et al., 2009; Suwa et al., 2010; Kim et al., 2011). Early loss of individual seed sink activity at high temperature may result from a reduction of the activity of starch synthesis-related enzymes in the seed (Ito et al., 2009; Suwa et al., 2010; Yamakawa and Hakata, 2010; Kim et al., 2011). At high temperature, synthesis of hemicelluloses, cellulose, and starch in grain declines while sucrose accumulates (Ito et al., 2009; Suwa et al., 2010; Yamakawa and Hakata, 2010). While increasing temperatures might impede phloem transport, they also might hasten the preferential unloading of carbon (C) along the stem to meet local increasing respiratory demand (Atkin and Tjoelker, 2003; Sevanto, 2014). The resulting enrichment in N relative to C in the phloem sap reaching the seeds would explain its higher N concentration (Layzell and Larue, 1982).

### Definition of Plant Senescence Under Heat Stress and Strategies to Develop Cultivars Adapted to Higher Temperatures Due to Climate Change

The original results of our study throw a new light on the regulation of N remobilization and definition of senescence in plants submitted to abiotic stress, such as heat-stress. At moderate temperatures senescence is linked to N remobilization to filling seeds, a mechanism to compensate the limitation of N uptake by roots (Hebbar et al., 2014). On the other hand, this research established that the heat-induced senescence (noticeable through the reduction of seed-filling duration) is surprisingly not associated with an acceleration of N nutrient remobilization to filling seeds. Under high temperature, shorter duration of seed-filling with increasing temperature may more likely result from alterations in various photosynthetic attributes and carbon budget than from plant N resources remobilization to cope with the heat stress (Wahid et al., 2007; Mathur et al., 2014).

Our results demonstrate that seed N yield processes are and will continue to be very frequently sink-limited by high temperatures during the seed-filling period in the warming climate context. It is worth noting that under the current and future climate change context, the increased frequency of early heat waves are and will be often associated to water deficit in field, resulting from either decreased precipitation and/or increased evaporation (Dai, 2013; Sehgal et al., 2018). The combined effects of water deficit and heat-stress on crops are more severe (Sehgal et al., 2018). Both abiotic constraints were previously reported to enhance assimilate remobilization from source to sink (Pic et al., 2002; Sehgal et al., 2018). On the contrary, our study using labeled nitrate demonstrates that N assimilate remobilization was reduced and most likely sink-limited under heat stress. Consequently, sustaining seed sink demand and preserving photosynthetic attributes of stressed plants during the seed-filling period should be promising strategies to maintain crop N production under exacerbated combined heat and water-deficit stresses in field due to the on-going climate change. Such improvements may especially require further investigations in order to elucidate how sink activity could be modulated at high temperature and water deficit. While water deficit can be mitigated by irrigation (Bueckert et al., 2015), few cultural practices are available to leverage high temperatures stress. A better understanding of mechanisms controlling C and N allocation to sinks, are required to build robust sustainable practices.

## Data Availability Statement

The datasets generated for this study are available on request to the corresponding author.

## Author Contributions

AL and NM-J designed the study. AL collected and analyzed the data. AL and NM-J interpreted the results. Both authors contributed to manuscript writing.

## Funding

This research was supported by INRA, AgroSupDijon and a grant of UNIP (Union Nationale Interprofessionnelle des Plantes Riches en Protéines).

## Conflict of Interest

The authors declare that the research was conducted in the absence of any commercial or financial relationships that could be construed as a potential conflict of interest.
