# Supplementary material for: High Temperatures During the Seed-Filling Period Decrease Seed Nitrogen Amount in Pea (Pisum sativum L.): Evidence for a Sink Limitation
Source: Front Plant Sci. 2019 Dec 20;10:1608. doi: 10.3389/fpls.2019.01608 (PMC6934051; doi:10.3389/fpls.2019.01608)
Supplement: Supplementary file 1 [file DataSheet_1.pdf]

SUPPLEMENTARY MATERIAL

TABLE S1. **Composition of the nutrient solutions.** In Exp. 1 and 2, pea plant nitrogen (N) nutrition relied exclusively on NO_3^- assimilation due to a high nitrate availability of the nutrient solution (14 meq NO_3^-). In Exp. 3, pea plant (N) nutrition relied exclusively on N_2 fixation due to a nutrient solution without nitrate (0 meq NO_3^-) and an inoculation with *R. leguminosarum*. Both nutrient solutions were produced by the greenhouse staff at INRA Dijon.

Nutrient element	Concentration of the nutrient element in the solution	
	**Exp. 1 & 2** (14 meq NO_3^-)	**Exp. 3** (0 meq NO_3^-)
	Mg l^{-1}	Mg l^{-1}
KNO_3	540.89	0.00
K_2HPO_4	34.84	139.34
$Ca(NO_3)^2$ +4H_2O	944.60	0.00
$MgSO_4$	90.28	120.37
$CaCl_2$	0.00	277.48
K_2SO_4	0.00	121.98
KH_2PO_4	122.48	0.00
$Mg(NO_3)^2$ +6H_2O	83.33	0.00
NaCl	11.69	11.69
H_3BO_3	2.00	2.00
$MnSO_4$+7H_2O	1.80	1.80
$ZnSO_4$+7H_2O	0.22	0.22
$H_{24}N_6O_{24}Mo_7$ + 4H_20	0.18	0.18
$CuSO_4$ + 5H_2O	0.08	0.08
EDTA FeNa+H_2O	20.66	20.66
EDTA FeNa	18.82	18.82

TABLE S2. **Yield (seed dry matter), vegetative dry matter (stems, leaves, roots and pods) and total dry matter of plants at maturity.** Pea plants were exposed to temperature treatments during the seed-filling period, i.e. from the beginning of seed filling of the last reproductive node (BSL) to plant maturity. Mean temperature during the seed-filling period (with standard error) was assessed as the average of the daily air temperatures observed from BSL to maturity (14-hour day length).

	N nutrition pathway	**Mean temperature during the seed-filling period (°C)**	Yield (Seed dry matter) (g plant^{-1})	Vegetative dry matter at maturity (g plant^{-1})	Total dry matter at maturity (g plant^{-1})
**Exp. 1**	$N0_3^-$ Assimilation	**18.4** (±0.2)	5.24 (±0.33)	8.02 (±1.23)	13.26 (±1.47)
		**23.2** (±0.1)	4.34 (±0.46)	7.60 (±1.03)	11.93 (±1.34)
		**28.1** (±0.6)	3.73 (±0.15)	5.39 (±1.52)	9.12 (±1.38)
		**33.2** (±0.4)	2.55 (±0.16)	5.23 (±0.64)	7.77 (±1.43)
**Exp. 2**	$N0_3^-$ Assimilation	**21.8** (±0.6)	2.78 (±0.22)	5.55 (±0.59)	8.32 (±0.66)
		**25.8** (±1.5)	2.45 (±0.15)	3.71 (±0.64)	6.16 (±0.51)
		**26.8** (±1.9)	2.22 (±0.20)	3.53 (±0.61)	5.75 (±0.71)
**Exp. 3**	N_2 Fixation	**19.9** (±0.5)	4.27 (±0.58)	4.20 (±0.50)	8.47 (±0.42)
		**26.1** (±0.6)	3.65 (±0.29)	4.04 (±0.47)	7.69 (±0.67)
		**28.6** (±0.8)	3.13 (±0.27)	4.86 (±0.30)	7.99 (±0.30)
		**31.3** (±1.5)	2.42 (±0.29)	4.67 (±0.23)	7.09 (±0.34)
